# Development of a Two-Stage Hydrometallurgical Process for Gold–Antimony Concentrate Treatment from the Olimpiadinskoe Deposit

**DOI:** 10.3390/ma16134767

**Published:** 2023-07-01

**Authors:** Rostislav Rusalev, Denis Rogozhnikov, Oleg Dizer, Dmitry Golovkin, Kirill Karimov

**Affiliations:** 1 NORD Engineering LLC, 119049 Moscow, Russia; rusalevrostislav@gmail.com; 2 Laboratory of Advanced Technologies in Non-Ferrous and Ferrous Metals Raw Materials Processing, Ural Federal University, 620002 Yekaterinburg, Russia; darogozhnikov@yandex.ru (D.R.); dmitry.golovkin@urfu.ru (D.G.); k.a.karimov@urfu.ru (K.K.)

**Keywords:** refractory gold ores, nitric acid, leaching, antimony, arsenic, gold cyanidation, optimization, mathematical planning of experiments

## Abstract

An integrated two-stage metallurgical process has been developed to process concentrates from the Olimpiadinskoe deposit, which contain high levels of antimony and arsenic. The optimal parameters for the alkaline sulfide leaching process of the initial concentrate from the Olimpiadinskoe deposit were determined to achieve the maximum extraction of antimony at a 99% level. The recommended parameters include an L:S ratio of 4.5:1, a sodium sulfide concentration of 61 g/L, a sodium hydroxide concentration of 16.5 g/L, a duration of 3 h, and a temperature of 50 °C. A synergistic effect of co-processing alkaline sulfide leach cakes with sulfuric and nitric acids was observed. The pre-treatment step reduced the nitric acid composition by converting carbonates into gypsum and increased the arsenic extraction by 15% during subsequent nitric acid leaching. The laboratory research on the nitric acid leaching of decarbonized cake established the key parameters for the maximum iron and arsenic extraction in solution (92% and 98%, respectively), including an L:S ratio of 9:1, a nitric acid concentration of 6 mol/L, and a time of 90 min. Full polynomial equations for the iron and arsenic extraction from the decarbonized cake were derived. The model demonstrated a high relevance, as evidenced by the determination coefficients (R^2^) of 96.7% for iron and 93.2% for arsenic. The technology also achieved a high gold recovery rate of 95% from the two-stage alkaline sulfide and nitric acid leach cake. Furthermore, the maximum deposition of arsenic from the nitrate leach solution in the form of insoluble As_2_S_3_ was determined to be 99.9%. A basic technological flow sheet diagram for processing the flotation gold–antimony concentrate from the Olimpiadinskoe deposit was developed, including two stages: the production of metallic antimony and the gold extraction from the nitric leach cake.

## 1. Introduction

Antimonite (Sb_2_S_3_) is the primary industrial mineral for antimony and is often associated with gold in various ways. Depending on the content of gold and antimony, the ores can be classified into different groups [1]. The selection of a suitable technology is determined by the predominant valuable compounds (gold or antimony) in the ore or concentrate. Pyrometallurgical technologies are primarily employed for sulfide–antimony ores and concentrates with a low arsenic content (up to 1%) and that contain gold [2]. The choice and justification of the pyrometallurgical technology primarily depend on the antimony content in the initial concentrate. Gold is considered a byproduct in the antimony refining process [3,4].

The presence of even small concentrations of antimony in gold-containing ores has a detrimental effect on the process of gold cyanidation. Antimonite undergoes oxidation, forming compounds such as antimonites (HSbO_3_^2−^), antimonates (HSbO_4_^2−^), thioantimonates (SbS_3_), and others. This adversely impacts the desired outcomes, including a low gold extraction into solution and an increased consumption of cyanide and lime [5,6]. Consequently, a preliminary preparation of the raw materials is required.

Currently, the prevalence of gold–antimony complex ores is growing and economically justified. However, the utilization of conventional extraction technologies for antimony and gold may result in significant losses of the second valuable component and incur high operational expenses. Therefore, processing these complex ores typically involves a sequential approach, starting with the removal of antimony materials followed by the gold extraction [7,8]. However, gold–antimony ores often exhibit a refractory behavior due to the association of gold with sulfides, such as antimonite (Sb_2_S_3_), pyrite (FeS_2_), and arsenopyrite (FeAsS). The presence of fine gold in rock-forming minerals is a common factor contributing to the refractoriness of such ores. Additionally, the significant content of arsenic, antimony, and iron compounds increases the operating costs associated with their extraction, requiring additional preparatory steps for cyanidation of the concentrate [9]. This restricts the application of existing processing methods for gold-bearing materials [10] and leads to the storage of these concentrates in specialized landfills or their sale to countries where their processing is possible due to lower environmental protection requirements.

The existing acidic methods employed for the extraction of antimony from gold–antimony ores have several drawbacks. These include a low antimony extraction into solution and the formation of elemental sulfur, which has a detrimental effect on the subsequent gold cyanidation processes [11].

It is important to highlight the promising nature of the alkaline sulfide method, which exhibits a high selectivity and the ability to process complex raw materials. This method enables the achievement of a high extraction of antimony into solution while minimizing environmental impacts. However, it should be noted that the current results obtained from the alkaline method cannot be directly applied to the processing of refractory gold-containing raw materials, where gold is closely associated with sulfide minerals. In practice, various technologies are employed for the sulfide matrix recovery, including oxidative roasting, ultra-fine grinding, pressure oxidation (POX), bioleaching (BIOX), nitric acid oxidation, and others [12,13].

The utilization of the current pyrometallurgical technologies is constrained due to the elevated toxicity of volatile arsenic compounds, necessitating additional expenditures for dust and gas cleaning systems. Consequently, this increases the capital requirements for production [14,15,16].

The implementation of hydrometallurgical methods [17,18,19,20,21,22,23] provides a solution for recycling low-grade raw materials, thereby addressing the disposal challenges associated with toxic elements and reducing the loss of valuable components through exhaust gases [24,25,26]. In Russia, the use of POX [27,28] and BIOX [29,30] methods have been explored. However, due to various limiting factors, including the complexity of the oxidation process and high capital requirements, the adoption of these processes by gold mining companies remains limited. Furthermore, these methods are not applicable to materials with a high antimony content, emphasizing the need for alternative technologies.

The nitric acid leaching technology [31,32,33,34] is emerging as a potential alternative to the existing methods. In this process, the sulfide material is treated with a solution containing nitric and/or nitrogen oxides (NSC) [35,36]. The exothermic reaction generates heat, thereby enhancing the oxidation process [37].

In light of the aforementioned factors, the objective of this research is to develop a synergistic combination of hydrometallurgical technologies for the recycling of gold–antimony concentrates, aiming to achieve a high extraction efficiency using environmentally sustainable approaches.

## 2. Materials and Methods

### 2.1. Analysis

Chemical analyses of the initial concentrate, alkaline leaching cakes, and nitric acid leaching cakes were performed using the Axios MAX X-ray fluorescence spectrometer (Spectris plc., London, UK). The gold content in the feedstock and leaching products was determined using an assay analysis and inductively coupled plasma mass spectrometry on a NexION 350D instrument (PerkinElmer Inc., Waltham, MA, USA). A phase analysis was carried out on the XRD 7000 Maxima diffractometer (Shimadzu Corp., Tokyo, Japan). The grain size and morphology of the resulting cakes were analyzed using a JEOL JSM-6390LA scanning electron microscope (JEOL Ltd., Tokyo, Japan) equipped with a JED-2300 energy dispersive analyzer. The chemical analysis of the solutions was determined through inductively coupled plasma mass spectrometry (ICP-MS) using the Elan 9000 instrument (PerkinElmer Inc., Waltham, MA, USA).

### 2.2. Materials and Reagents

**Figure 1:**
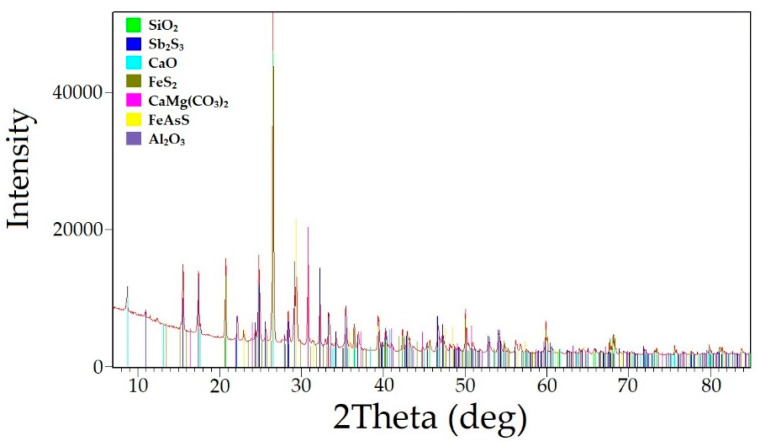
Radiograph of the gold–antimony concentrate phase composition.

The main raw material used in this study was the flotation concentrate obtained from the Olimpiadinskoe deposit located in Krasnoyarsky Krai, Russia. The concentrate consisted of a quartz antimonite semi-sulfuride rock. The chemical composition of the concentrate is presented in Table 1, and the phase composition is shown in Figure 1. Based on the results, it was determined that the main minerals in the studied raw material were quartz (SiO_2_) at 33.6%, antimonite (Sb_2_S_3_) at 26.7%, dolomite (CaMg(CO_3_)_2_) at 5.3%, calcium oxide (CaO) at 15.0%, pyrite (FeS_2_) at 5.7%, arsenopyrite (FeAsS) at 4.7%, and corundum (Al_2_O_3_) at 3.1%.

**Figure 2:**
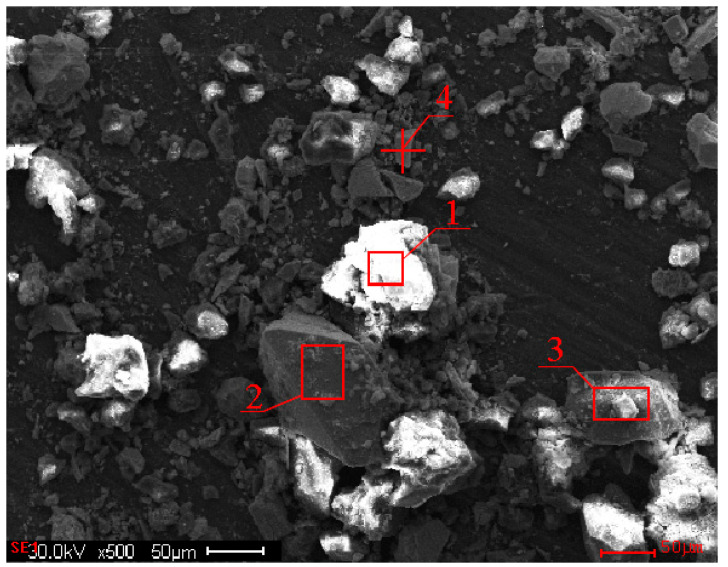
A microphotograph of the universal particles of the initial concentrate at the Olimpiadinskoe deposit.

As shown in Figure 2 and Table 2, the results of the study of the compositions of the individual grains of the initial sulfide concentrate are shown.

Based on the data presented in Table 2, Area 1 exhibited a predominance of Sb_2_S_3_ and FeS_2_, with sulfur (S) accounting for 22.29%, antimony (Sb) for 48.65%, and iron (Fe) for 12.10%. In addition to antimonite and pyrite, the presence of SiO_2_, CaO, and a probable association of gold with sulfides (Au 1.4%) was observed (O: 8.65%, Si: 1.36%, Ca: 4.14%).

Area 2, representing the grain mass of the initial concentrate, was primarily composed of the following elements: 18.81% As, 10.63% Sb, and 39.97% Fe. The presence of 6.79% O and 3.21% Si in this area suggested the presence of silicon dioxide (SiO_2_). Furthermore, the occurrence of 0.92% Au indicated a possible association with sulfides.

Area 3 was characterized by the prevailing elements S (28.80%), Sb (11.72%), Fe (36.39%), and O (10.41%). It contained Sb_2_S_3_ and FeS_2_ minerals, with the probable presence of Fe_2_O_3_, SiO_2_, and CaO.

In the spectrum obtained from point four, the peaks corresponding to Sb (67.64%) and S (22.65%) confirmed the presence of Sb_2_S_3_ in this area. Additionally, the presence of Au (2.34%) suggested an association with sulfide minerals.

### 2.3. Experimental Procedure

#### 2.3.1. Alkaline Sulfide and Nitric Acid Leaching

The laboratory experiments involving alkaline sulfide and nitric acid leaching were conducted under atmospheric pressure in a thermostatted glass reactor with an outer jacket, specifically the Lenz Minni-60 (Lenz Laborglas GmbH & Co. KG, Wertheim, Germany), with a volume of 500 cm^3^. The experiments were carried out at temperatures of 50 °C and 80 °C, respectively. Mixing was performed using a top-mounted agitator operating at 400 rpm. The material sample was added to water and heated to the desired temperature, after which the alkali or acid was gradually added. At the end of the experiment, the leach pulp was filtered using a Buchner funnel (ECROSKHIM Co., Ltd., St. Petersburg, Russia). The resulting leach cake was then washed with distilled water, dried at 80 °C until a constant mass was achieved, ground using a planetary mill Pulverisette 6 classic line (Fritsch GmbH & Co. KG, Welden, Germany), pressed onto a backing plate using the hydraulic Vaneox 40t Automatic (Fluxana GmbH & Co. KG, Bedburg-Hau, Germany), and sent for XRF analysis. The obtained solutions were diluted to the required concentrations of Fe, As, and Sb in measuring flasks with volumes of 25, 50, and 100 dm^3^, respectively, using a 1% nitric acid solution and subsequently sent for analysis.

The calculations of free Gibbs energy values and Eh-pH charting were carried out using the HSC Chemistry Software v. 9.9 (Metso Outotec Finland Oy, Tampere, Finland).

#### 2.3.2. Electroextraction and Smelting of Cathode Antimony

The electrolysis cell was comprised of individual cathode chambers connected in series with a current source. The containers were constructed using steel grade DIN 17100, and each container had a useful volume of 2.5 dm^3^. The anode was constructed from a steel rod and fully immersed in the reactor. It was positioned perpendicularly to the bottom of the cathode container, which had a cover composed of polymethyl methacrylate. The electric supply to the cell was provided by a rectifier transformer unit. At the end of the process, the cathode antimony, along with the electrolyte, was separated using a Nutch filter. The cathode sediment was subsequently washed and dried. The spent electrolyte was analyzed to determine the presence of ballast salts and the concentration of antimony.

The dried cathode sediment was blended with technical soda and sodium hydroxide at a 1:1 ratio. The resulting mixture was placed into a crucible and subjected to a controlled smelting process using a laboratory muffle furnace (Nabertherm L 3/11, Nabertherm GmbH, Lilienthal, Germany) at a temperature of 1000 °C for a duration of 80 min. Following the smelting process, the molten material was allowed to cool naturally to room temperature, facilitating the mechanical separation of the resulting metallic antimony and slag. The obtained metallic antimony was then carefully collected and prepared for X-ray fluorescence (XRF) analysis to determine its elemental composition.

#### 2.3.3. Decarbonization

The presence of 15% dolomite (CaMg(CO_3_)_2_) and 16% CaO in the cakes resulting from alkaline leaching led to a significant increase in the consumption of nitric acid. To address this, a decarbonization operation was employed to remove these components.

Sulfuric acid was utilized in an open glass container at room temperature (25 °C). The mixing process was conducted using an agitator at a speed of 400 rpm. Prior to the addition of sulfuric acid, the material was pulped with water, maintaining a liquid-to-solid (L:S) ratio of 4:1 (here and throughout the text, the L:S ratio is considered as a volume-to-mass ratio). Sulfuric acid was gradually added until the emission of carbon dioxide ceased. Following the completion of the process, the resulting pulp was subjected to filtration. The obtained cakes were thoroughly washed with distilled water, dried at 80 °C until a constant mass was achieved, and subsequently sent for X-ray fluorescence (XRF) analysis.

#### 2.3.4. Arsenic Precipitation

Following the nitric acid leaching process, the resulting solutions were subjected to arsenic precipitation in the form of As_2_S_3_. The pH variations in the system were monitored using a universal pH meter (Seven2Go, Mettler Toledo, Columbus, OH, USA). A filtrate volume of 70 cm^3^ was transferred to a 200 cm^3^ glass vessel and thoroughly mixed. Sodium hydrosulfide (NaHS) with a concentration of 72 g/L was added to initiate the sulfuric–arsenic precipitation process, which lasted approximately one hour as determined in a previous study [26]. After the precipitation process, the pulp was filtered, and the obtained sediment was dried at 60 °C until a constant mass was achieved. The dried sediment was subsequently sent for X-ray fluorescence (XRF) analysis. The remaining solution after precipitation was analyzed to determine the residual arsenic content.

#### 2.3.5. Cyanidation of the Nitric Acid Leach Cake

A plastic container was used to hold a leach solution with a concentration of NaCN (2 g/L) and NaOH (2 g/L). A portion of the nitric leach cake weighing 100 g was added to the container. The liquid-to-solid ratio (L:S) was maintained at 3:1 with a pH value of 11. After a 24-h period, the pulp was filtered, and the resulting cake was thoroughly washed with distilled water until a neutral washing solution was obtained. The washed cake was then dried, weighed, and sent for gold assay analysis.

## 3. Results and Discussion

### 3.1. Theoretical Overview of Sulfide–Alkaline Leaching

Antimony, due to its amphoterism, exhibits solubility in both acidic and alkaline solutions. However, in industrial settings, a water mixture containing sodium sulfide and caustic soda is commonly employed for the dissolution of antimonite. Alkaline solutions comprising sodium sulfide serve as a versatile and selective solvent for most antimony minerals [38,39,40], while the dissolution of arsenic, tin, and mercury minerals proceeds at a relatively slower pace [40,41,42]. The dissolution reaction of antimonite can be represented by Equation (1).

(1) $${\mathrm{Sb}}_{2}S_{3}{+3\mathrm{Na}}_{2}{S=2\mathrm{Na}}_{3}{\mathrm{SbS}}_{3(\mathrm{liq})};\;∆G_{25}^{0}=-71.41\;\mathrm{kJ}/\mathrm{mol}$$

Sodium hydroxide (NaOH) plays a significant role in the dissolution of antimony, as it prevents the hydrolysis of sodium sulfide (Na_2_S). The reaction proceeds in two stages, as described by Equations (2) and (3) [39].

(2) $${\mathrm{Na}}_{2}{S+H}_{2}O=\mathrm{NaHS}+\mathrm{NaOH},\;∆G_{25\;}^{0}=-66.99\;\mathrm{kJ}/\mathrm{mol}$$

(3) $${\mathrm{NaHS}+H}_{2}{O=H}_{2}S+2\mathrm{NaOH},\;∆G_{25\;}^{0}=-79.65\;\mathrm{kJ}/\mathrm{mol}$$

The total reaction is as follows (Equation (4)).

(4) $${\mathrm{Na}}_{2}{S+2H}_{2}{O=H}_{2}S+2\mathrm{NaOH},\;∆G_{25\;}^{0}=-8\;\mathrm{kJ}/\mathrm{mol}$$

In cases where there is an insufficient amount of sodium sulfide, which is the primary solvent in the Na_2_S and NaOH mixture, sodium hydroxide (NaOH) serves not only as a hydrolysis inhibitor for Na_2_S but also as an additional solvent for antimony. This is demonstrated by the following reaction (Equation (5)) [38].

(5) $${\mathrm{Sb}}_{2}S_{3}{+6\mathrm{NaOH}=\mathrm{Na}}_{3}{\mathrm{SbS}}_{3}{+\mathrm{Na}}_{3}{\mathrm{SbO}}_{3}{+3H}_{2}O,\;∆G_{25\;}^{0}=-67\;\mathrm{kJ}/\mathrm{mol}$$

In addition to its role as a solvent for antimony, sodium sulfide can react with oxygen and carbon dioxide that are present in the atmosphere [39]. These reactions can be represented by Equations (6) and (7).

(6) $${\mathrm{Na}}_{2}{S+3/2O}_{2}{=\mathrm{Na}}_{2}{\mathrm{SO}}_{3},\;∆G_{25\;}^{0}=-155\;\mathrm{kJ}/\mathrm{mol}$$

(7) $${2\mathrm{Na}}_{2}{S+H}_{2}{O+\mathrm{CO}}_{2}{=2\mathrm{NaHS}+\mathrm{Na}}_{2}{\mathrm{CO}}_{3},\;∆G_{25\;}^{0}=-49\;\mathrm{kJ}/\mathrm{mol}$$

Equations (1) and (5) describe the dissolution of antimonite in alkaline sulfide solutions. However, the undesirable side reactions six and seven occur simultaneously, resulting in the formation of unwanted ballast salts that can reduce the process efficiency. Another notable reaction occurring in the solution is the formation of thiosulfates, which are structural analogues of sulfates with one oxygen atom replaced by a sulfur atom. Thiosulfates exhibit unique complexing abilities [43]. The formation of thiosulfates can be represented by the following reaction (Equation (8)).

(8) $${2\mathrm{Na}}_{2}{S+2O}_{2}{+H}_{2}{O=\mathrm{Na}}_{2}S_{2}O_{3}+2\mathrm{NaOH},\;∆G_{25\;}^{0}=-200\;\mathrm{kJ}/\mathrm{mol}$$

Thiosulfate compounds, similar to the cyanide process, form strong complex bonds with gold, as represented by reaction nine [43]. The interaction between gold and thiosulfate ions results in the formation of a stable complex, [Au(S_2_O_3_)_2_]^3–^, which remains intact upon acidification without a sulfur release. The dissolution of gold in a thiosulfate solution in the presence of oxygen can be described by the following reaction (Equation (9)).

(9) $${4\mathrm{Au}+8\mathrm{Na}}_{2}S_{2}O_{3}{+O}_{2}{+H}_{2}{O=4\mathrm{Na}}_{3}\left[\mathrm{Au}{\left(S_{2}O_{3}\right)}_{2}\right]+4\mathrm{NaOH},\;∆G_{25\;}^{0}=-468\;\mathrm{kJ}/\mathrm{mol}$$

**Figure 3:**
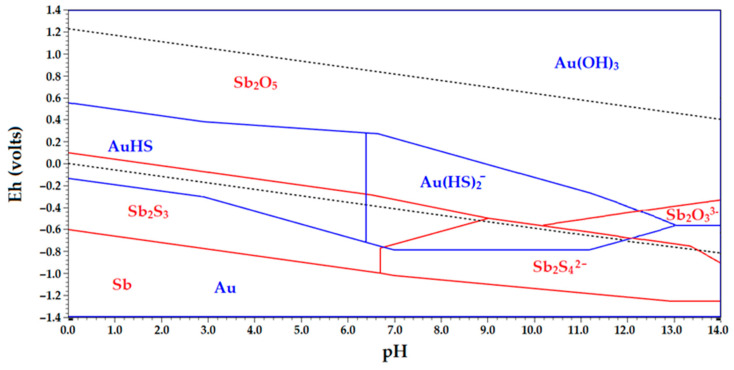
Eh-pH diagram for the Au–Sb–S–H_2_O system at 25 °C.

Figure 3 presents the Eh-pH diagram illustrating the stable forms of the Sb–Au–S–H_2_O system. The thermodynamic data indicates that gold can form soluble compounds, namely Au(HS)^2−^ and AuS^−^, within a pH range of 0 to 13. Beyond a pH of 13, gold will no longer dissolve into the solution. In the alkaline region, under negative potentials, the solution contains a complex ion [Sb_2_S_4_]^2−^ as well as a complex ion of a complete oxidant [Sb_2_O_3_]^3−^ [44].

The Eh-pH diagram shows the stability region of strong Sb_2_S_3_ at a molar concentration ratio of (Sb)/(S) = 1/3, as shown on the opposite side of the diagram. According to the data from references [44,45], when the (Sb)/(S) ratio decreased to 0.25 or lower, a significant portion of the Sb_2_S_3_ region represented in the diagram disappeared, and the region corresponding to water-soluble Sb compounds expanded. This suggested that the most effective leaching of antimony from its mineral occurs at a (Sb)/(S) ratio of ≤0.25. Conversely, increasing this ratio enhanced the potential for antimony electrodeposition, which was advantageous for the subsequent electrolysis of antimony from sulfide electrolytes. However, a practical challenge arises due to the requirement for excess sulfide ions for the efficient antimony dissolution from the minerals, while an excess of free sulfide ions hinders the electrolysis process and reduces the antimony current output [40].

### 3.2. Results of the Refined Antimony Production

#### 3.2.1. Alkaline Sulfide Leaching

To determine the parameters of alkaline sulfide leaching, a central composite design (CCD) with three variable parameters was conducted, consisting of seventeen experiments. The software Statgraphics v. 18 was employed for the data analysis. A full quadratic model was selected to process the experimental results, with the extraction of antimony in the solution serving as the response variable. The chosen variables included the liquid-to-solid (L:S) ratio in the pulp, ranging from 1.2 to 6.7, and the concentrations of NaOH and Na_2_S, ranging from 16.4 to 43.5 g/L and 16.2 to 61.1 g/L, respectively. The leaching time and temperature were maintained at 180 min and 50 °C, respectively. These ranges were determined based on the preliminary test experiments and relevant literature sources [46,47]. The results of the variance analysis are shown in Table 3.

Based on the data presented in Table 3, it can be inferred that the L/S ratio and Na_2_S concentration exhibited a significant statistical influence on the alkaline sulfide leaching process for antimony.

**Figure 4:**
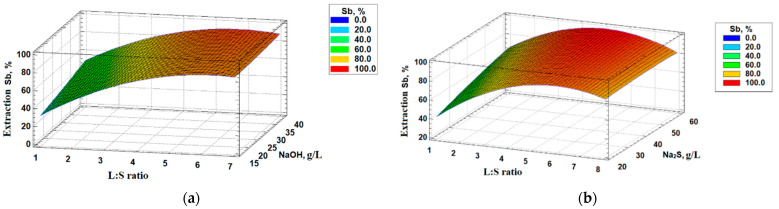
Dependencies of antimony extraction (**a**) on the L:S ratio and concentration NaOH and (**b**) on the L:S ratio and concentration Na_2_S.

The obtained response surfaces (Figure 4) illustrated the variation in antimony recovery across different solvent concentrations and L/S ratios. It was observed that increasing the L/S ratio led to a maximum extraction of antimony, with an optimal ratio of 4.5:1. However, beyond this point, further increases in the L/S ratio resulted in a decrease in antimony extraction. Moreover, antimony extraction showed an upward trend as the solvent concentration increased.

The obtained results, represented by a complete polynomial equation (Equation (10)), provided a means to evaluate the influence of each factor on antimony extraction (U_Sb_).

(10) $$U_{\mathrm{Sb}\;}=1.03+24.5X+0.44Y+1.81Z\;-\;0.03\mathrm{XY}\;-\;0.08\mathrm{XZ}\;-\;0.15\mathrm{YZ}\;-\;1{.94X}^{2}+0{.004Y}^{2}-\;0{.01Z}^{2}$$

where X is the L:S ratio, Y is the concentration of NaOH g/L, and Z is the concentration of Na_2_S, g/L.

The coefficient of determination, R^2^, was calculated to be 95.4%. This high value indicated that the chosen quadratic model and regression equation were suitable for describing the data accurately.

Based on the obtained results, the optimal parameters for the alkaline sulfide leaching process of the initial concentrate, resulting in a maximum antimony extraction in the solution, were determined as an L:S ratio of 4.5:1, a sodium sulfide concentration of 61 g/L, a sodium hydroxide concentration of 16.5 g/L, a leaching time of 3 h, and a temperature of 50 °C. These parameters yielded a remarkable antimony extraction of 99%.

**Figure 5:**
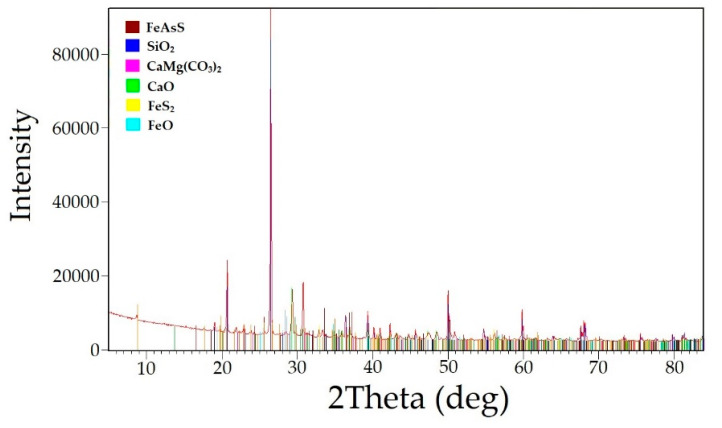
The diffractogram of the cake after alkaline sulfide leaching.

The chemical composition of the leached cake after alkaline sulfide leaching is presented in Table 4. Additionally, the phase analysis radiograph is shown in Figure 5.

The analysis of the leach cakes revealed that they mainly consisted of quartz (SiO_2_—35.41%), dolomite (CaMg(CO_3_)_2_—15.17%), calcium (CaO—16.34%), pyrite (FeS_2_—7.53%), arsenopyrite (FeAsS—7.17%), and iron oxide (FeO—6.1%).

**Figure 6:**
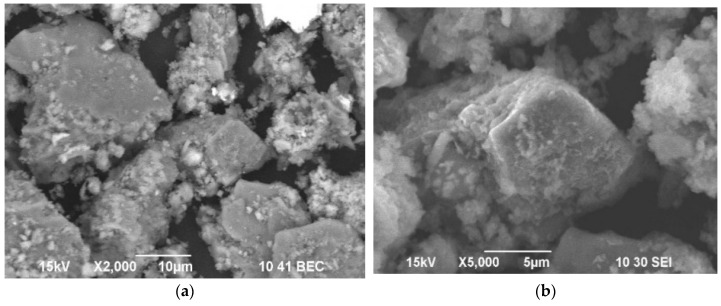
Individual particles after alkaline sulfide leaching at (**a**) 2000 times and (**b**) 5000 times magnification.

Furthermore, Figure 6 illustrates the surface characteristics of the cake particles, revealing the presence of pores and caverns. These surface features were attributed to the influence of the alkaline sulfide solution during the leaching process.

#### 3.2.2. Antimony Electroextraction 

For the electrolysis process, an 8.5 dm^3^ volume of a productive solution containing 30 g/L of antimony was used. The electrolysis conditions involved a cathode current density ranging from 140 to 150 A/m^2^, an anode current density ranging from 900 to 1000 A/m^2^, a bath voltage between 2.4 and 4.0 V, an amperage of 12 A, a duration of 16 h, and an electrolyte temperature of 50–55 °C. These ranges were determined based on the preliminary test experiments and findings from the previous studies [48]. To enhance the process efficiency, alkaline and sodium sulfide were added to the electrolyte at concentrations of 30 and 60 g/L, respectively.

At the end of the electrolysis, the antimony cathode, along with the electrolyte, was separated using a Nutsche filter. The cathode sediment underwent washing and drying procedures. The spent electrolyte was analyzed for the presence of salts and antimony. The weight of the obtained antimony cathode was 183.6 g. The residual antimony content in the electrolyte was found to be 8.4 g/L. The current output reached 63.1%, while the efficiency was calculated to be 11.4 g/(m^2^ h). The power consumption for the process was determined to be 4190 kWh/t.

#### 3.2.3. Refining of Cathode Antimony

The dried cathode was mixed with technical soda (57.35 g—Na_2_CO_3_) and sodium hydroxide (22.94 g—NaOH) at a 1:1 ratio. The resulting mixture was placed in a crucible. The mixture was smelted in an induction furnace at a temperature of 1000 °C for a duration of 80 min [49].

After the smelting process, the melt was allowed to cool to room temperature. The slag was then mechanically separated from the antimony. The metallic antimony obtained had a mass of 73.98 g, while the slag weighed 86.62 g. The detailed results of the antimony refining process are presented in Table 5.

The refined antimony obtained from the process conformed to the grade 2N, which made it suitable for the production of various alloys, such as antifriction alloys, battery alloys, typographic alloys, and alloys used for cable sheaths [48,50].

### 3.3. Obtaining Gold-Bearing Residue Applicable for Cyanidation

#### 3.3.1. Thermodynamic Analysis of Nitric Acid Leaching

The principal reactions occurring during the treatment of the dis-antimoniated cake with nitric acid at a temperature of 80 °C are depicted in Equations (11)–(14).

(11) $${2\mathrm{FeS}}_{2}{+8\mathrm{HNO}}_{3\;}{=\mathrm{Fe}}_{2}{{(\mathrm{SO}}_{4})}_{3\;}{+S}^{0}{+8\mathrm{NO}+4H}_{2}O;\;∆G_{80}^{0}=-1615\;\mathrm{kJ}/\mathrm{mol}$$

(12) $${\mathrm{FeS}}_{2}+6{\mathrm{HNO}}_{3}=\mathrm{Fe}({\mathrm{NO}}_{3}{)}_{3}+2S^{0}+3{\mathrm{NO}}_{2}+3H_{2}O;\;∆G_{80}^{0}=-257\;\mathrm{kJ}/\mathrm{mol}$$

(13) $${2\mathrm{FeAsS}+10\mathrm{HNO}}_{3}{+O}_{2}=2\mathrm{Fe}{\left({\mathrm{NO}}_{3}\right)}_{3}{+2H}_{3}{\mathrm{AsO}}_{4}{+2S}^{0}{+4\mathrm{NO}+2H}_{2}O;\;∆G_{80}^{0}=-1408\;\mathrm{kJ}/\mathrm{mol}$$

(14) $${3\mathrm{FeAsS}+23\mathrm{HNO}}_{3\;}{=3\mathrm{Fe}(\mathrm{NO}}_{3}{)}_{3}{+3H}_{3}{\mathrm{AsO}}_{4}{+3H}_{2}{\mathrm{SO}}_{4}{+14\mathrm{NO}+4H}_{2}O;\;∆G_{80\;}^{0}=-3146\;\mathrm{kJ}/\mathrm{mol}$$

The oxidation of the sulfides exhibited a notable thermo-effect. Reaction (11) represented the predominant reaction during the oxidation of pyrite. This reaction occurred rapidly at temperatures exceeding 60 °C and pH values below 1.7 [37]. When the concentration of nitric acid was high (above 50 g/L), a small quantity of elemental sulfur was formed even at relatively low temperatures. Conversely, at lower concentrations of nitric acid, the production of elemental sulfur increased, particularly at low temperatures [31].

Arsenopyrite exhibited a higher reactivity compared to pyrite, making its oxidation likely even at room temperature, depending on the concentration of nitric acid. The decomposition of arsenopyrite in nitric acid resulted in the formation of elemental sulfur (Equation (13)).

**Figure 7:**
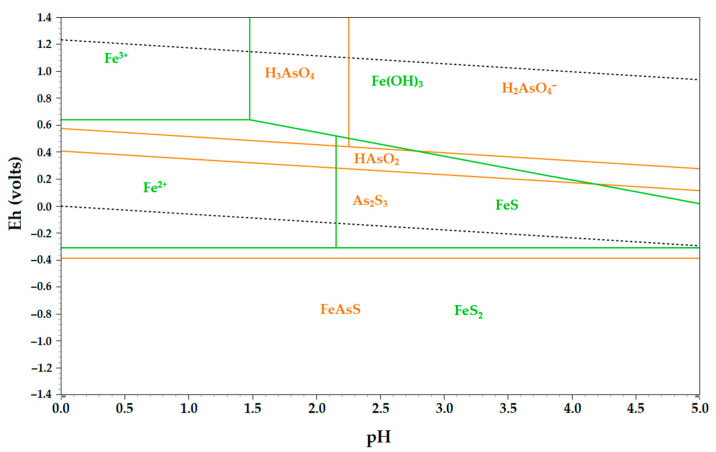
Eh-pH diagram for the Fe–As–S–N–H_2_O system.

To evaluate the thermodynamic behavior of pyrite and arsenopyrite in nitric acid, an Eh-pH diagram was constructed (Figure 7).

In the Fe-S-N-H_2_O system, it was observed that arsenopyrite began to dissolve when the system potential reached 0.39 V, resulting in the formation of arsenic sulfide. As the system potential further increased to around 0.4 V, arsenic converted to meta-arsenic acid, and upon reaching approximately 0.6 V, it transformed into ortho-arsenic acid. On the other hand, pyrite initiated the decomposition at a potential value of −0.3 V, leading to the formation of Fe^2+^ cations and FeS. As the potential exceeded 0.63 V, iron (II) cations transitioned to the form Fe (III).

#### 3.3.2. Decarbonization

For the purpose of comparing the efficiency of nitric acid leaching for the obtained materials (dis-antimoniated and decarbonized cakes), a series of experiments were conducted using the following parameters: a nitric acid concentration of 265–900 g/L, an L:S ratio of 5–16:1, and a leaching duration of 80–150 min.

The results of the nitric acid leaching experiments are presented in Table 6. The data indicated that the extraction of iron and arsenic into the solution was higher when the decarbonized cake was leached. During the leaching of the cake, nitric acid was primarily utilized to dissolve CaO and CaMg(CO_3_)_2_, which in turn reduced the oxidizing potential of the system.

The decarbonization, on the other hand, enabled the decomposition of dolomite into CaSO_4_, MgSO_4_, and CO_2_. Calcium sulfate remained in the cake and did not hinder the nitric acid leaching process.

**Figure 8:**
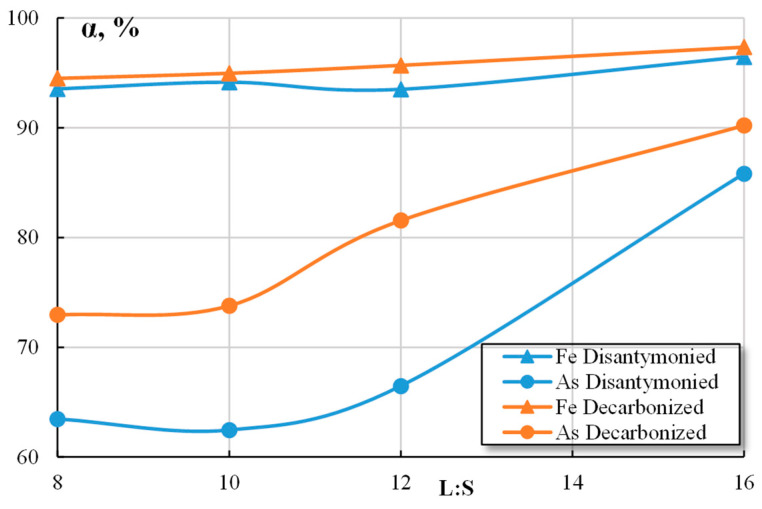
Dependences of the Fe and As extraction on the L:S ratio.

The dependencies of iron and arsenic extraction into the solution using a nitric acid concentration of 600 g/L and a leaching time of 80 min are shown in Figure 8. Increasing the L:S ratio from eight to 16 had a minimal effect on the iron transfer into the solution. The extraction rates increased from 93.52% to 96.45% for the dis-antimoniated cakes and from 94.5% to 97.33% for the decarbonized cake. However, the extraction of arsenic into the solution was significantly higher during the leaching of the decarbonized cake. As the L:S ratio increased, the extraction of arsenic also increased from 72.95% to 90.2%. Without additional processing using sulfuric acid, the maximum extraction of arsenic did not exceed 85.81%.

The composition of the dis-antimoniated cake after the sulfuric acid treatment is presented in Table 7.

#### 3.3.3. Results of Optimizing the Parameters of Nitric Acid Leaching of the Decarbonized Cake

**Figure 9:**
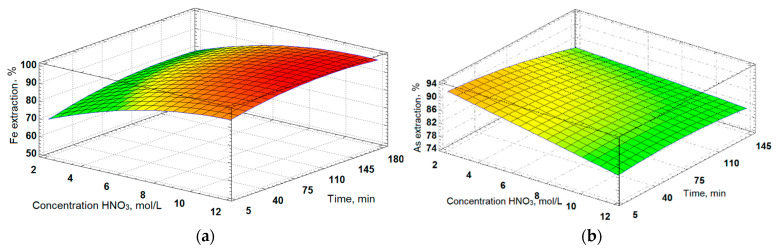
Dependencies of (**a**) the iron extraction at the L:S ratio 2:1 and (**b**) the arsenic extraction at the L:S ratio 9:1 on the time of the experiments and acid consumption.

The parameters of nitric acid leaching were determined using the Statgraphics software v. 18 through a composition plan consisting of 17 experiments with three changeable parameters. A full quadratic model was applied to process the obtained results. The response surfaces were generated and are illustrated in Figure 9. A general regression equation was derived, and the determination coefficients R^2^ were calculated. The nitric acid concentration varied from 2.5 to 13.4 mol/L, the L:S ratio ranged from 2.3 to 22:1, and the leaching time was adjusted between 8 and 171 min. These ranges were selected based on the preliminary test experiments. The variance analysis results are presented in Table 8.

Based on the data presented in Table 8, it can be concluded that the nitric acid concentration and leaching time were highly statistically significant factors affecting the iron extraction. On the other hand, the L/S ratio was the statistically significant parameter affecting the arsenic extraction. The obtained results, presented as a complete polynomial, allowed for the assessment of the influence of each factor on the extraction of iron (U_Fe_) and arsenic (U_As_).

(15) $$\;{\;U}_{\mathrm{Fe}}=48.9+1.14X+5.73Y+0.14Z\;-\;0.03\mathrm{XY}+0.0004\mathrm{XZ}\;-\;0.002\mathrm{YZ}\;-\;0{.02X}^{2}-\;0{.21Y}^{2}-\;0{.0004Z}^{2}$$

(16) $$\;\;\;U_{\mathrm{As}}=64.3+2.27X+1.03Y+0.01Z\;-\;0.09\mathrm{XY}+0.002\mathrm{XZ}+0.005\mathrm{YZ}\;-\;0{.04X}^{2}-\;0{.003Y}^{2}-\;0{.0002Z}^{2}$$

where X is the value of the L:S ratio,

Y is the concentration HNO_3_, mol/L,

Z is the leaching time, min.

The determination coefficients (R^2^) for iron and arsenic were found to be 96.7% and 93.2%, respectively, indicating the adequacy of the chosen full quadratic model and the derived regression equation.

**Figure 10:**
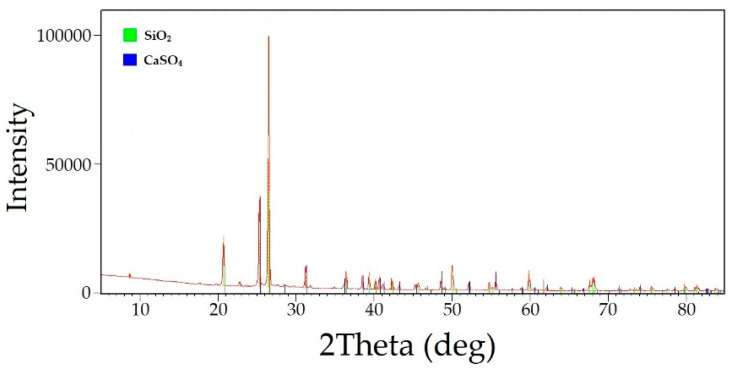
The radiograph of the undissolved residue after nitric acid leaching.

Based on these results, the optimal parameters for nitric acid leaching of the decarbonized sulfide leach cakes were determined to be an L:S ratio of 9:1, a nitric acid concentration of 6 mol/L, and a leaching time of 90 min. At these parameters, the iron and arsenic extraction efficiencies reached 98% and 92%, respectively. The chemical composition of the resulting leach cake is provided in Table 9. The XRD analysis (Figure 10) revealed the presence of anhydride (CaSO_4_) and quartz (SiO_2_) as the main phases in the residue. 

#### 3.3.4. Results of Arsenic Precipitation from the Productive Nitric Acid Leaching Solution

In the one-stage process of nitric acid leaching of the decarbonized cake, the resulting solution contained free nitric acid, which negatively affected the subsequent stage of arsenic precipitation. This led to an increased reagent consumption. To address this issue and improve the utilization of nitric acid, an additional portion of the cake was added in excess at a temperature of 85 °C, continuing the leaching process until the emission of nitrous gas was completed. This addition stimulated the second stage of the leaching process.

The resulting solution obtained after this step exhibited iron and arsenic concentrations of 41.5 g/L and 23.4 g/L, respectively. Arsenic precipitation was conducted based on the previous research [26] under specific conditions: a stoichiometric flow rate of NaHS/As of 1.6 and a pH of 1.96. Following the sedimentation process, the pulp underwent metabolic reactions between iron and arsenic for 10–20 min. The extraction of arsenic into the sediment reached up to 99.9%.

#### 3.3.5. Gold Cyanidation

The results of cyanidation of the nitric acid leach cake are presented in Table 10. The gold extraction efficiency into the solution was determined to be 95%. The cyanide consumption during the process was measured to be 6 kg /t.

#### 3.3.6. The Principal Technological Flowsheet of Gold–Antimony Concentrate Recycling at the Olimpiadinskoe Deposit

**Figure 11:**
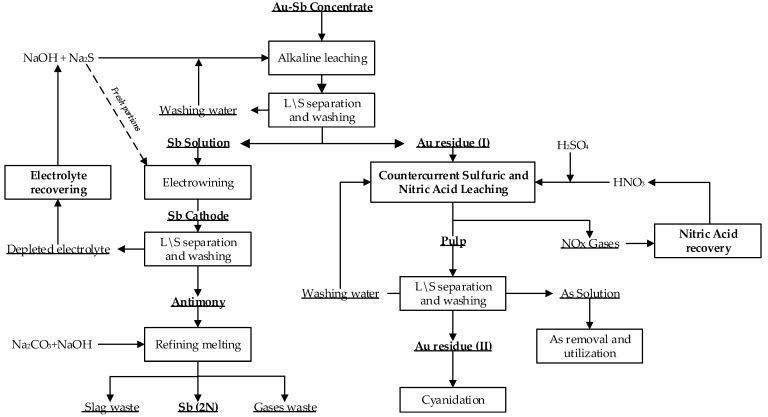
The principal technological flowsheet for processing the Au–Sb concentrate at the Olimpiadinskoe deposit.

The laboratory studies yielded valuable data, enabling the development of a fundamental technological flowsheet for the processing of refractory complex gold–antimony concentrates sourced from the Olimpiadinskoe deposit. The proposed flowsheet is depicted in Figure 11.

In order to enhance the effectiveness of alkaline sulfide leaching, the process was implemented in a closed-loop system. The antimony extraction was performed using recycled solutions and a reconditioned electrolyte. Periodically, a portion of the solution was removed to eliminate impurities, and then reintroduced into the circulation. Purge water was utilized for the reconstitution of new batches of concentrates and the adjustment of the sulfide–alkaline solutions.

The cake obtained after alkaline sulfide leaching was subjected to a two-stage counter-current sulfuric–nitrate leaching process. In the first stage, decarbonization is carried out using sulfuric acid, while leaching occurred with the enriched solution from the second leaching stage. This facilitated the neutralization of excess nitric acid and increased the regeneration of nitric acid. In the second leaching stage, the pre-oxidation of sulfides took place in a reactor using more concentrated nitric acid solutions (5 mol/L). The generated nitric oxides were directed towards nitric acid regeneration. The resulting insoluble sediment was filtered and washed, and the wash water was utilized for the nitric acid regeneration and/or for preparing a new pulp for a new batch of cake. The washed cake containing gold was subsequently subjected to cyanidation, while the filter was used for arsenic precipitation following the first leaching stage.

## 4. Conclusions

Comprehensive two-stage hydrometallurgical technology has been developed for processing gold-containing concentrates with high concentrations of antimony and arsenic from the Olimpiadinskoe deposit. The research findings have led to the following conclusions.

To achieve a maximum antimony extraction into the solution during alkaline sulfide leaching and facilitate the transition of gold into the cake, it was necessary to maintain a pH level above 13. This ensured the prevention of sulfide ion dissociation from the gold particles present in the concentrate, thus enhancing the efficiency of the antimony extraction.

Based on the study, the recommended parameters for the alkaline sulfide leaching process of the initial concentrate from the Olimpiadinskoe deposit—to achieve a 99% maximum antimony extraction into the solution—are as follows: a liquid-to-solid ratio (L:S) of 4.5:1, a sodium sulfide concentration of 61 g/L, a sodium hydroxide concentration of 16.5 g/L, a leaching time of 3 h, and a temperature of 50 °C.

The synergetic effect resulting from the combined treatment of sulfide–alkaline leach cakes with sulfuric and nitric acids was successfully demonstrated. The pre-treatment of the leach cakes with sulfuric acid was found to have significant benefits, including a reduction in nitric acid consumption and an increase in arsenic extraction during the subsequent nitric acid leaching stage by approximately 15%.

The laboratory tests on the nitric acid leaching of the decarbonated cake identified the main parameters for extracting iron and arsenic into the solution, which were found to be 98% and 92%, respectively. The optimized parameters for achieving these extraction rates were an L:S ratio of 9:1, a nitric acid concentration of 6 mol/L, and a leaching time of 90 min. Complete polynomial equations for the iron and arsenic extraction from the decarbonized cake were obtained, and the adequacy of the model was confirmed by the coefficient of determination, with R^2^ values of 96.7% for iron and 93.2% for arsenic. Additionally, a high gold extraction rate of 95% was observed in the cake during the two-stage alkaline sulfide and nitric acid leaching process.

A comprehensive technological flowchart was developed for the initial processing of the gold–antimony concentrate from the Olimpiadinskoe deposit. The flowchart comprised two distinct processes: the metallic antimony extraction and the gold extraction from the nitric acid leaching cake.

The first step involved an alkaline sulfide leaching process, followed by electrolytic antimony extraction and refining the cathode precipitate. This process yielded metallic antimony with a purity level of 2 N.

The second step encompassed the decarbonization of the alkaline sulfide leach cake, which was followed by a counter-current nitric acid leaching process that facilitated nitric acid regeneration. During this stage, the solutions were acidified, leading to the formation of a relatively insoluble arsenic sulfide (As_2_S_3_) precipitate, which was safely disposed of. Finally, gold was extracted from the resulting cakes obtained after the nitric acid leaching process.

## Figures and Tables

**Table 1:** Chemical composition of the gold–antimony concentrate.

Element	As	Ca	Fe	Mg	S	Sb	Si	Al	Others	Au (g/t)
Wt.%	1.9	13.4	8.95	1.46	11.46	19.18	18.6	1.69	23.36	66.00

**Table 2:** The study results of the individual grains of the initial concentrate from the Olimpiadinskoe deposit.

Element	Area 1		Area 2		Area 3		Point 4	
	Wt. %	At. %	Wt. %	At. %	Wt. %	At. %	Wt. %	At. %
O K	8.65	26.63	6.79	19.34	10.41	25.14	2.48	9.97
As L	1.42	0.93	18.81	11.44	1.64	0.84	0.4	0.34
Si K	1.36	2.38	3.21	5.21	1.63	2.25	1.48	3.38
Au M	1.40	0.35	0.92	0.21	1.16	0.23	2.34	0.76
S K	22.29	34.25	17.03	24.2	28.8	34.69	22.65	45.39
Sb L	48.65	19.69	10.63	3.98	11.72	3.72	67.64	35.69
Ca K	4.14	5.09	2.64	3.00	8.26	7.96	2.21	3.54
Fe K	12.10	10.67	39.97	32.62	36.39	25.17	0.80	0.92

**Table 3:** The variance analysis of antimony leaching.

Source	Sum of Squares	Df	Mean Square	F-Ratio	*p*-Value
A: L/S ratio	820.985	1	820.985	51.78	0.0002
B: NaOH concentration	42.5848	1	42.5848	2.69	0.1453
C: Na_2_S concentration	735.979	1	735.979	46.42	0.0003

**Table 4:** Chemical composition of the cake after alkaline sulfide leaching.

Element	As	Ca	Fe	Mg	S	Sb	Si	Others	Au (g/t)
Wt.%	1.73	12.4	6.84	1.54	6.84	0.08	18.6	39.67	78.00

**Table 5:** Antimony melting results, Wt.%.

Product Name	Sb	S	As	Fe	Al	Ca	Si
Antimony cathode	92.1	2.6	0.73	3.35	0.23	0.1	0.95
Refined antimony	99.759	0.05	0.02	0.03	0.02	0.05	0.1
Slag	0.24	2.37	0.66	3.08	0.20	5.04	0.80

**Table 6:** The results of nitric acid leaching of the decarbonized and dis-antimoniated cakes.

No	HNO_3_, g/L	L:S	Duration, min	Extraction, %	
				Fe	As
Decarbonized cakes					
1	265	5	150	93.11	77.62
2	630	5	120	92.17	69.78
3	900	7	150	96.23	72.11
4	600	8	80	94.5	72.95
5	600	10	80	94.36	73.78
6	600	12	80	95.68	81.55
7	600	16	80	97.33	90.2
Dis-antimoniated cakes					
8	265	5	150	86.42	68.49
9	630	5	120	90.58	46.41
10	900	7	150	90.66	52.72
11	600	10	80	94.13	62.45
12	600	8	80	93.52	63.46
13	600	12	80	92.49	66.46
14	600	16	80	97.45	85.81

**Table 7:** Chemical composition of the decarbonized cake.

Element	As	Ca	Fe	S	Sb	Si	Others
Wt.%	3.40	14.80	11.10	19.00	4.40	16.40	30.9

**Table 8:** The variance analysis results of antimony leaching.

Source	Sum of Squares	Df	Mean Square	F-Ratio	*p*-Value
Iron extraction					
L/S ratio	0.164925	1	0.164925	0.07	0.7955
Nitric acid concentration	57.7287	1	57.7287	25.37	0.0015
Leaching time	54.4569	1	54.4569	23.93	0.0018
Arsenic extraction					
L/S ratio	2.01178	1	2.01178	12.29	0.0099
Nitric acid concentration	0.007006	1	0.007006	0.04	0.8420
Leaching time	0.10917	1	0.10917	0.67	0.4411

**Table 9:** Chemical composition of the nitric acid leach cake.

Element	As	Ca	Fe	K	Mg	S	Sb	Si	O	Au (g/t)
Wt.%	0.39	11.3	0.25	0.58	0.12	14.5	3.14	16.20	54.22	101.40

**Table 10:** Gold cyanidation results.

Material	Au	
	g/t	Extraction, %
Cake after HNO_3_	101.4	0
Cake after cyanidation	5.07	95
